# Allergic Conjunctivitis Management: Update on Ophthalmic Solutions

**DOI:** 10.1007/s11882-024-01150-0

**Published:** 2024-06-13

**Authors:** Andrea Leonardi, Luigi Quintieri, Ignacio Jáuregui Presa, Jesús Merayo LLoves, Jesús Montero, José Manuel Benítez-del-Castillo, Francisco Javier Seoane Lestón, Eloína González-Mancebo, Riccardo Asero, Anna Groblewska, Piotr Kuna

**Affiliations:** 1 Department of Neurosciences & Ophthalmology, University of Padua, Padua, Italy; 2 Laboratory of Drug Metabolism, Department of Pharmaceutical and Pharmacological Sciences, University of Padua, Padua, Italy; 3 Allergy Department, Hospital Universitario de Cruces, Bilbao, Spain; 4 Instituto Universitario Fernández-Vega, University of Oviedo, Asturias, Spain; 5 Department of Surgery, University of Seville, Seville, Spain; 6 CARTUJAVISION Eye Clinic, Seville, Spain; 7 Department of Ophthalmology, Hospital Clínico San Carlos, Universidad Complutense, Clínica Rementería Madrid, Madrid, Spain; 8 Department of Allergy, Hospital Severo Ochoa de Leganés, Madrid, Spain; 9 Unit of Allergy. Hospital Universitario de Fuenlabrada, Madrid, Spain; 10 Department of Allergy, Clinica San Carlo, Paderno Dugnano, Milan, Italy; 11 Ophthalmology Department, Polish Mother’s Memorial Hospital, Lodz, Poland; 12 Department of Internal Medicine, Asthma and Allergy, Medical University od Lodz, Lodz, Poland

**Keywords:** Allergic Conjunctivitis, Topical Treatment, Antihistamines, Preservatives, Benzalkonium Chloride (BAK), Hyaluronic acid (HA)

## Abstract

**Purpose of Review:**

The aim of this review, is to present an updated revision of topical management of SAC and PAC, based on the available scientific evidence and focused on the impact of ophthalmic solution formulations on eye surface.

**Recent Findings:**

Physicians treating ocular allergy should be aware of tear film and tear film disruption in SAC and PAC, and how eye drop composition and additives affect the physiology of the allergic eye.

**Summary:**

Seasonal and perennial allergic conjunctivitis (SAC and PAC) are the most frequent causes of ocular allergy (OA), and both conditions are underdiagnosed and undertreated. SAC and PAC are immunoglobulin E (IgE)-mediated hypersensitivity reactions. The additional tear film disruption caused by the release of inflammatory mediators increases and exacerbates the impact of signs and symptoms and may trigger damage of the ocular surface. Comorbidities are frequent, and dry eye disease in particular must be considered. Clinical guidelines for the management of SAC and PAC recommend topical therapy with antihistamines, mast cells stabilizers or dualaction agents as first-line treatment, but care should be taken, as many medications contain other compounds that may contribute to ocular surface damage.

## Introduction

Ocular allergy (OA) is a common immunological inflammatory process of the anterior surface of the eye [1]. In fact, OA represents a collection of underestimated diseases of the eye observed in children and adults that present with a wide spectrum of clinical forms [2].

Allergic conjunctivitis (AC) is the most frequent OA and affects the ocular surface for the duration of aeroallergen exposure. Therefore, its nosology includes seasonal allergic conjunctivitis (SAC) and perennial allergic conjunctivitis (PAC), depending on whether the specific aeroallergen is seasonal or permanent [1]. Grass pollen is the most common allergen in SAC, while dust mites and animal dander are the most frequent sensitization in PAC; food allergens may also occasionally cause ocular symptoms [1, 3]. AC is associated with an immunoglobulin E (IgE)-mediated mechanism also referred to as Th2-mediated inflammation [2, 4, 5].

The prevalence of OA has been increasing worldwide for several decades [6]. SAC and PAC can affect between 14% and up to 45% of the general population, depending on geographical region [6]. However, AC is an often underdiagnosed and undertreated health problem, with only 10% of patients with AC symptoms seeking medical attention: most sufferers manage their condition with over-the-counter (OTC) medications and complementary, non-pharmacological remedies [1]. SAC is by far the most common form of OA, representing over half of all cases [1]. Furthermore, OA commonly overlaps with other ocular disease disorders, including infections and dry eye disease (DED) [1]. OA also has a significant impact on quality of life (QoL) and carries a high economic burden [7].

Successful management of OA involves preventive, non-pharmacological, and pharmacological measures [1]. Over the years, various therapeutic options have been developed to achieve symptom control. First-line options are topical antihistamines, mast cell stabilizers, and dual-acting agents, while corticosteroids and immunomodulators are reserved for severe cases of keratoconjunctivitis [4, 5, 8]. It should be kept in mind that many patients with OA are treated with topical treatments over long periods of time [7] and, therefore, the short- and long-term safety and tolerability of products used to treat the eye surface are paramount. OA is treated by general practitioners, allergists, and ophthalmologists, and all these professionals should be aware of the benefits and potential harms of the products prescribed. For instance, some compounds found in ophthalmic solutions may induce damage to the ocular surface by the disruption of epithelial barrier of tear film, which can lead to other diseases such as dry eye, or could exacerbate SAC and PAC [8, 9].

The purpose of this review is to present updated information on the management of OA for healthcare providers seeing these patients, with a particular focus on topical treatments for SAC and PAC.

## Problems Associated with Allergic Conjunctivitis

The pathophysiology of AC is complex, and the condition is often accompanied by other problems than should be borne in mind during treatment, such as tear film disruption and concomitant disorders. Awareness of these aspects will help decision-making.

### Tear Film Disruption

Epithelial barrier dysfunction is a hallmark feature of several ocular allergic disorders [8] and tear film alterations are often observed in patients with AC. The eye presents some physiological barriers, including complex junctions in conjunctival and corneal epithelium [8] that help it act as a physical and immune barrier, preventing the entrance of pathogens and allergens and helping maintain tissue homeostasis. In AC, allergens with intrinsic proteolytic activity cross the conjunctival epithelium and enter the submucosal space, where they interact with dendritic and conjunctival mast cells to induce allergic inflammation with activation of inflammatory cytokines such as interleukin 33 (IL-33), thymic stromal lymphopoietin (TSLP), and transforming growth factor (TGF)-β [8]. The disrupted corneal epithelium can also release neuromediators, including substance P (SP), calcitonin gene-related peptide (CGRP), vasoactive intestinal peptide (VIP), and nerve growth factor (NGF) [8]. These soluble mediators activate dendritic cells (DC) to generate a typical Th2 lymphocyte response in SAC and PAC [2]. The subsequent sensitization and activation of adaptive immune response leads to type 2 inflammation characteristic of the allergic disorders. In SAC and PAC, exposure of sensitized patients to the allergen results in a type I IgE-mediated allergic reaction, that provokes mast cell degranulation and the immediate release of histamine.

Overexpression of Th2-associated cytokines (IL4, IL-5, IL-13) and histamine contribute to long-term conjunctival epithelial metaplasia, disruption of the corneal epithelium and stimulation of goblet cell secretion, which could modify the tear film [10].

In addition to the impact of AC on ocular surface, some of ophthalmic solutions for its treatment frequently contain compounds and preservatives that may contribute to the disruption of epithelial barrier of tear film [8, 9]. The most common preservative is benzalkonium chloride (BAK), and its impact on the ocular surface has been extensively studied [10]. BAK is a detergent [11] that disrupts the tear film and damages the epithelial barrier, exerting a pro-inflammatory action through activation of the nuclear factor kappa B (NF-kB) pathway [10]. In vitro studies and murine models [12, 13] suggest that prolonged exposure to BAK leads to ocular damage due to a loss of immune tolerance of the conjunctival epithelium of the eye.

### Concomitant Pathologies and Differential Diagnosis

Allergic conjunctivitis is often comorbid with allergic rhinitis (AR) [14]. The two disorders are so strongly and frequently associated that they are defined in most countries as a single condition (e.g., allergic rhinoconjunctivitis) [15]. When this happens, conjunctival symptoms are often perceived by clinicians or even patients as a minor problem, and therefore ocular manifestations are frequently misdiagnosed or underdiagnosed, and not properly managed [1, 16] or sub-optimally treated [10]. Therefore, when compared to AR, allergic conjunctivitis receives far less attention [7]. In many countries, most cases are diagnosed and managed by a general practitioner and rarely referred to specialists, even when other signs and symptoms suggest a systemic allergic disorder that would be more appropriately treated by a specialist [1]. Notably, the therapeutic approach of primary care physicians and eye care specialists has been discordant [1], and this is also true for allergists. In this regard, a common knowledge base such as that provided by this review may be useful to detect areas for improvement.

DED and AC coexist and show a significant clinical overlap. In fact, AC and DED are the most common ocular surface disorders, and they both have a potentially severe impact on patients’ QoL [15]. Bearing DED in mind is crucial when assessing AC as they can both be considered as conditions that predispose to or at least facilitate each other [10]. Eye itching, conjunctival redness and tear film dysfunction have been detected in both conditions, thus complicating differential diagnosis [10]. In addition, the prolonged use of some topical and systemic medications, especially eye drop formulations containing BAK as a preservative, can contribute to the development of DED.

The diagnosis of AC is based on clinical history, signs and symptoms, and confirmed with in vivo and in vitro allergen testing [16]. An exhaustive ocular examination to assess abnormalities of the conjunctiva, cornea, and tear film, together with collection of the patient’s clinical history should always be the first steps. Allergen-specific IgE response should be ascertained: the skin prick test (SPT), along with in vitro specific IgE measurement, is still considered the gold standard for this, although if the relationship between allergen exposure and ocular signs and symptoms is not clear, a conjunctival allergen provocation test may also be performed [1, 17].

### Over-the-counter Medications

One important problem is that patients with ocular allergic disease often self-medicate; they purchase OTC medications and fail to seek help even when those therapies are ineffective [22, 23]. In one study [18], self-treatment measures were the first step taken by 56% of patients diagnosed with AC. Many OTC drugs are topical vasoconstrictors or decongestants that have limited efficacy in OA, as they do not treat the underlying cause of the disease [7]. Conversely, the use and overuse of OTC products may lead to adverse effects such as rebound vasodilation [23]. Furthermore, BAK is a very common preservative in these solutions – present in over 70% of OTC eye drops – that, as mentioned, has the potential to induce ocular toxicity and exacerbate ocular surface damage [7].

## Is There Room for Improvement in the Management of AC?

AC continues to be an underdiagnosed and undertreated disease [7]. Considering the need for differential diagnosis, a multidisciplinary approach involving allergists, general practitioners, otolaryngologists, and eye care specialists is advisable [1, 17]. It should be borne in mind that in many settings, allergic conjunctivitis patients are often seen in primary care and, when referred to a specialist, those with SAC symptoms are usually referred to the allergist, while patients with PAC are more often referred to the ophthalmologist for differential diagnosis [1]. Therefore, inter-consultation channels or appropriate cross-referral between specialists (allergists and eye care specialists) is advisable, as fluent communication between disciplines would optimize patient care and improve outcomes [1].

Finally, it is necessary to increase understanding and awareness about the impact of preservatives and other potentially harmful compounds in ophthalmic topical treatments. Anti-allergic eye drops that maintain ocular surface homeostasis while avoiding the toxic effects of preservatives should be considered as standard of care, especially if long-term treatment is expected.

## Update on Topical Pharmacological Treatment

The recommended management approach for acute and chronic forms of OA starts with allergen identification, followed by non-pharmacological treatments (allergen avoidance and hygiene measures), progressing finally to pharmacological treatment [1]. Etiological treatment with various forms of immunotherapy can be offered under the guidance of specialists [1, 19]; when specific sensitization is the main cause of ocular allergy, allergen immunotherapy should be considered, as it has shown to be effective in reducing total and individual ocular symptoms of SAC and PAC [1, 17, 19].

The aim of pharmacological treatment in OA is to provide control and relief of signs and symptoms [1]. According to guidelines and consensus documents, topical antihistamines, mast cell stabilizers or double-action drugs should be the first line of treatment for the management of SAC and PAC [1, 4, 17, 19]. Several studies have focused on the safety differences between these different topical treatment groups in terms of the potential damage on ocular surface or their role in the development of conditions such as DED [9, 20–23]. Summaries of the characteristics of approved products are listed in Tables 1 and 2. When symptoms of allergic rhinitis are present, treatment options usually include systemic antihistamines and intranasal corticosteroids [1]. In this scenario, the use of second-generation systemic antihistamines, with fewer adverse events and a much better profile in terms of sedation, is recommended [4, 5, 7]. The potential of some systemic antihistamines to worsen the ocular surface due to their anticholinergic effect should be taken into account [10]. Systemic leukotriene receptor antagonists have proved to be useful in the treatment of allergic rhinitis, although less than oral antihistamines [17]; but they have a limited use for the treatment of OA [1, 24]. Topical immunomodulators such as calcineurin inhibitors, cyclosporine and tacrolimus, are recommended for chronic diseases of OA (vernal or atopic keratoconjunctivitis) [1, 17]. Their use depends on severity and frequency of exacerbations. Topical corticosteroids may be used for severe forms of OA and in uncontrolled exacerbations of SAC and PAC since their use is associated with potentially significant adverse reactions [17, 25].

**Table 1:** Characteristics of topical multi-dose antihistamines and dual-action agents for the treatment of SAC and PAC

**Topical ophthalmic solutions**	**Active ophthalmic solution**	**Dose (mg/ml)**	Inactive ingredients (excipients) *	**Co-formulation with HA**	Daily dose *	**Preservatives (%)**
**Antihistamines**	Bilastine [64]	0.6 mg/ml	• hydroxypropyl-β • cyclodextrin • methyl cellulose • sodium hyaluronate • anhydrous glycerine • sodium hydroxide 1n • water for injection	Yes	Once daily	Free
	Cetirizine [70]	2.4 mg/ ml	• glycerine • sodium phosphate • dibasic • edetate disodium • polyethylene glycol 400 • polysorbate 80 • hypromellose • hydrochloric acid/ sodium hydroxide (to adjust pH) • water for injection	No	Three times daily	BAK 0.01
	Emedastine [71]	0.5 mg/ml	• tromethamol • sodium chloride • hypromellose • purified water • hydrochloric acid and/or sodium hydroxide (to adjust ph).	No	Twice daily	BAK 0.01
	Levocabastine [72]	0.5 mg/ml	• propylene glycol • disodium phosphate anhydrous • monobasic sodium phosphate monohydrate • hypromellose • polysorbate 80 • edetate calcium disodium (e385)	No	Twice daily	BAK 0.15
**Dual-action agents**	Alcaftadine [73]	2.5 mg/ml	• edetate disodium • monobasic sodium • phosphate • purified water • sodium chloride • sodium hydroxide • hydrochloric acid (to adjust pH)	No	Twice daily	BAK 0.05
	Azelastine [74]	0.5 mg/ml	• hypromellose • disodium edetate • liquid sorbitol (crystallising) • sodium hydroxide (to adjust pH) • water for injection	No	Twice daily	BAK 0.125 Available Free [55, 75]
	Bepostatine besilate [76]	15 mg/ml	• sodium phosphate • sodium chloride • sodium hydroxide • water	No	Twice daily	BAK 0.05
	Epinastine [77]	0.5 mg/ml	• disodium edetate • sodium chloride • sodium dihydrogen phosphate dihydrate • sodium hydroxide/hydrochloric acid (to adjust pH) • purified water	No	Twice daily	BAK 0.1
	Ketotifen [78, 79]	0.025 mg/ml 0.05 mg/ml	• glycerol (e422) • sodium hydroxide (e524) • water for injections	No	Twice daily	BAK 0.1 Available free [48]
	Olopatadine [80, 81]	1 mg/ml [80] 2.22 mg/ml [81]	• sodium chloride • disodium hydrogen phosphate dodecahydrate (e339) • hydrochloric acid (e507) • sodium hydroxide (e524) • purified water	No	Twice daily Once daily	BAK 0.1 Available free [49]

*BAK* benzalkonium chloride, *HA* hyaluronic acid, *OS* ocular surface

* Described in technical data sheet

**Table 2:** Clinical efficacy and safety of topical multi-dose approved antihistamines and dual action agents for treatment of SAC and PAC

	**Active ophthalmic solution**	**Clinical trials**	**Efficacy**	Safety: OS adverse events *	**Considerations**
	Bilastine [31, 35, 36, 39, 64]	NCT03231969; NCT03479307 PMID: 35,234,641 PMID: 36,811,846 PMID: 36,909,350	Superior compared with vehicle Non-inferior compared with ketotifen [31, 38, 82]. Superior to vehicle [31, 38]	Adverse reactions are uncommon and include • dry eye • eye discharge • eye irritation • lacrimation • increased ocular discomfort	Approved by EMA
	Cetirizine [33, 34, 70]	NCT01881113 PMID: 30,587,908 PMID: 30,858,690	Superior compared with vehicle [33, 34]	Common side effects include: • blurry vision • eye burning/stinging upon instillation • eye pain • lid oedema • keratitis • hyperaemia	Approved by FDA
	Emedastine [71]	CAC : C-93-19 CAC: C-94-90. NCT00133627	NCT0013362 (in addition to ketotifen) [83]	Common adverse events: • eye pain • itchy eyes • redness of the eyes	Approved by FDA and EMA
	Levocabastine [72]	PMID: 8,101,534 [84] PMID: 7,902,024	Inferior compared with olopatadine [28] Superior compared with lodoxamide [85]	Common adverse events: • eye irritation • pain • swelling • itching • redness • burning sensation • watery eyes • blurred vision	Approved by FDA and EMA • Only in France approved Levocabastine preservative-free [86]
**Dual-action agents**	Alcaftadine [73]	PMID:29,543,548 [87] PMID:33,463,568 [45]	Superior to vehicle and non-inferior to olopatadine 0.1% [87] Bepotastine and alcaftadine appear to outweigh olopatadine in resolving the symptoms of allergic conjunctivitis [45]	Common side effects include • eye irritation, • burning and/or stinging upon instillation • eye redness • eye pruritus	Approved by FDA
	Azelastine [74]	PMID: 9,061,218 [88] PMID: 8,977,510 [89] PMID:12,841,925 [90] PMID:19,668,586 [91]	Superior to placebo [88] Superior to placebo [89] Non-inferior to levocabastine [90] Slightly inferior to olopatadine [91]	Common side effects include mild, transient irritation in the eye.	Approved by FDA and EMA • In Europe, azelastine preservative-free in single-dose [50] and multi-dose dispenser [55, 75]
	Bepostatine besilate [76]	NCT00586664 [92] NCT01861522 [93]	Superior to vehicle [92] Superior to placebo Safety in children between 7–15 years [93]	Common side effects include • taste perversion and bad taste • eye irritation	Approved by FDA
	Epinastine [77]	PMID: 14,996,516 [94] PMID: 14,996,515 [95] PMID: 12,841,924 [96] PMID: 18,691,985 [97]	Superior to vehicle [94, 95] and to placebo and to sodium cromoglycate [96] More comfortable than ketotifen and azelastine [97]	Common side effects include • burning sensation • eye irritation	Approved by FDA and EMA
	Ketotifen [78]	PMID:30,303,746 [98] PMID: 18,631,332 [99]	Superior: ketotifen 0.025% more effective and better tolerated compared with ketotifen 0.05% [98] Superior compared with fluorometholone acetate [99]	Common side effects include: • eye irritation • eye pain • punctate keratitis • punctate erosion of corneal epithelium	Approved by FDA and EMA • In Europe, ketotifen preservative-free in single-dose [47, 54] and multi-dose dispenser [48].
	Olopatadine [80, 81]	NCT01109485	Superior to ketotifen and superior to levocabastine [99–102]	Common side effects include • eye pain • eye irritation • dry eye • abnormal sensation in eyes	Approved by FDA and EMA • Olopatadine 2.22 mg/ml is approved by FDA and is an OTC • Olopatadine 1 mg/ml is approved by EMA • Olopatadine 0.77 once daily is approved by FDA • Olopatadine preservative-free in multi-dose dispenser [49]

*OS* ocular surface

* Described in technical data sheet

Topical non-steroidal anti-inflammatory drugs (NSAIDs) are not recommended for the treatment of SAC and PAC [17]. Many patients also require the concomitant use of lubricating eye drops to maximize control of ocular allergy symptoms [26].

Finally, in patients with severe forms of SAC and PAC, following the recommendations of the EAACI guidelines, allergen immunotherapy (AIT) should be considered a therapeutic option [19]. AIT is indicated when IgE-mediated hypersensitivity is evidenced, after first-line treatment failure, or to modify the natural course of ocular allergic disease. Systemic immunosuppressive treatment and biologic therapy may be prescribed in severe forms of OA, such as refractory vernal or atopic keratoconjunctivitis [17].

### Topical Antihistamines

Compared with oral antihistamines, topical antihistamines directly target ocular tissues and have a faster onset of action, good tolerance, and a better safety profile due to a lower systemic exposition [7]. Topical ophthalmic formulations containing second-generation antihistamines include levocabastine, emedastine, together with the most recent approvals, cetirizine and bilastine. Emedastine has shown to be superior to levocabastine [27], while olopatadine [28] has shown to be superior to emedastine [29, 30], and similar in terms of symptom reduction to some of the so-called dual-action agents. As mentioned above, two new topical antihistamines have recently been approved for the treatment of SAC and PAC: cetirizine, approved by the Food and Drug Administration (FDA) in the USA (not available in Europe at present), and a topical bilastine formulation [31] approved by the European Medicines Agency (EMA) in July 2022 (Tables 1 and 2).

An ophthalmic formulation with cetirizine 2.4 mg/ml evaluated efficacy compared to its vehicle (clinical trial NCT01881113). The cetirizine-containing eye drops reduced ocular itching and conjunctival redness significantly compared to vehicle, they were well tolerated, and showed an acceptable safety profile [32–34]. This cetirizine ophthalmic formulation needs to be used three times a day, as the dosage is one drop in each affected eye every 8 h. It contains BAK (0.01%) as a preservative to guarantee the sterility of the multi-dose formulation.

As for the newest bilastine ophthalmic solution, efficacy, tolerability, and efficacy of a once-daily administration have been evaluated in clinical trials to date [31, 35–39]. The bilastine formulation has shown a fast onset of action and efficacy for as long as 16 h post-administration, indicating that it is suitable for a once-daily dosing [31, 38]. In a phase three clinical trial, it also showed similar efficacy to a marketed multi-dose formulation of ketotifen 0.025% [35, 39]. This newly EMA-approved bilastine formulation is a preservative-free solution co-formulated with hyaluronic acid, unique in its class, presented in a special multi-dose container. Preclinical in vitro and in vivo studies have shown that these two characteristics (preservative-free with hyaluronic acid) help preserve the tear film and ocular surface integrity [31, 38].

### Topical Mast Cell Stabilizers

The mechanism of action by which topical mast cell stabilizers inhibit mast cell degranulation remains unclear [7]. Available mast cell stabilizers include lodoxamide, nedocromil or sodium cromoglycate, and others. However, recent studies [7, 26] suggest that mast cell stabilizers are not as effective in treating ocular allergies because to achieve their maximum effect they need to be used as a prophylactic measure prior to allergen exposure, thus decreasing compliance and adherence as compared to other anti-allergic ophthalmic agents [26, 40].

### Topical Dual-Activity Agents

Topical dualactivity agents (antihistamine/mast cell stabilizing activity) have been well studied and are supported by extensive clinical experience [7]. Although topical antihistamines and dual-activity agents are treated as a separate category in this article, recent publications include both antihistamines and these topical drugs in the same group, since at the conjunctival concentrations reached in direct topical use, almost all antihistamine eye drops also act as mast cell stabilizers to some extent [41, 42]. Examples of widely used topical dual-activity agents include azelastine, epinastine, ketotifen, olopatadine, alcaftadine, and bepotastine besilate (Tables 1 and 2). In terms of efficacy, olopatadine, ketotifen, alcaftadine and bepotastine have a similar efficacy profile [43, 44], although some studies reported that bepostatine and alcaftadine appear to be superior to olopatadine [45].

In the management of SAC, formulations containing ketotifen 0.025% have proven to be effective, although in a study that collected patient-reported measures of efficacy and comfort, patients preferred an olopatadine 0.1% solution [46]. Scientific evidence suggests that in terms of clinical relief and tolerability, dual-action agents are superior to antihistamines such as levocabastine [28] and mast cell stabilizers such as lodoxamide or cromolyn, although recent studies indicated that many have secondary effects, such as eosinophil migration inhibition and activation of cytokines and other inflammatory mediators [7]. Moreover, treatment with these therapeutic agents alone is usually not enough to control the condition [17]. Most of the commercialized formulations containing so-called dual-action agents also include BAK, which may cause ocular surface toxicity. In some European countries [47], preservative-free single-dose and multi-dose formulations containing ketotifen [47, 48], olopatadine [49] and azelastine [50] are available.

### Topical Vasoconstrictors

Topical ophthalmic vasoconstrictors, such as naphazoline, oxymetazoline, phenylephrine, and tetrahydrozoline, are α-adrenergic agonists that relieve conjunctival reddening caused by vasodilation [4]. They do not reduce the allergic response as they do not antagonize any of the mediators of the allergic reaction and inflammation, and instead only alleviate hyperaemia. Prolonged use may cause rebound hyperaemia and tachyphylaxis and consequently, these products should be used with caution and only for short periods of time [1, 17].

### Topical Ophthalmic and Intranasal Corticosteroids

Topical ophthalmic corticosteroids are rarely needed for the treatment of SAC and PAC, but may be used in acute exacerbations as short, pulsed therapy [17]; particularly in severe forms of OA such as vernal or atopic keratoconjunctivitis. The potency and duration of treatment should be clinically determined based on the severity of ocular inflammation and corneal involvement [17]. Despite being the most effective anti-inflammatory drugs in clinical practice for OA [17], they are associated with a myriad of potentially severe adverse reactions (increased intraocular pressure, cataract development, delayed wound healing, and increased susceptibility to infection or superinfections), so their use must be closely monitored by an ophthalmologist, especially in long-term treatments. They should be limited to the most severe forms of OA and severe and uncontrolled exacerbations [1, 17, 51].

Regarding intranasal corticosteroids, there is increasing evidence indicating that they are effective in reducing ocular symptoms associated with allergic rhinitis, so their use is recommended in the presence of comorbidities [1, 7, 17]. Intranasal steroids such as fluticasone furoate and mometasone furoate have shown positive effects on ocular allergic symptoms as compared to placebo [7, 52].

### Topical NSAIDs

NSAIDs block the cyclooxygenase enzyme and the conversion of arachidonic acid into prostaglandins, therefore reducing inflammation and signs and symptoms of AC. Ketorolac was the first approved for OA; however, like other topical ophthalmic NSAIDs, it was associated with discomfort on instillation, possibly affecting patient compliance. Topical ophthalmic NSAIDs are therefore not recommended for the management of SAC and PAC, due to significant irritation associated with instillation and other side effects, such as corneal melting and perforation. They are limited to the most severe forms of OA or for exacerbations that cannot be controlled by other measures, and always for as short a duration as possible [1].

## Considerations when Selecting an Ophthalmic Formulation

### Presence of Preservatives

A recurring concern when designing an ophthalmic drug is the need to include preservative compounds in multi-dose formulations to provide antimicrobial activity and ensure sterility [53]. It has been widely demonstrated that preservatives have a toxic effect on the ocular surface, and they lead to epithelium disruption and a tear film dysfunction [2, 8]. Many kinds and natures of preservatives are used in ophthalmic solutions, such as detergents (BAK), ionic buffers (propylene glycol), alcohols and parabens, among others. Nevertheless, BAK, present in approximately 70% of ophthalmic formulations, is the most common. The cytotoxic effects of BAK on ocular tissue cells have been extensively documented, and the estimated threshold at which toxicity occurs is at a concentration of 0.005% [53]. Multi-dose topical ophthalmic medications for the treatment of SAC and PAC containing BAK as a preservative usually exceed that threshold. Until now, preservative-free ophthalmic solutions were most often available in single-use (single-dose dose) presentations [50, 54], although nowadays multi-dose presentations, azelastine [53, 55], ketotifen [48], olopatadine [49] and the new bilastine eye drop formulation are BAK-free (Table 1). Notably, the absence of preservatives in ophthalmic solutions has been shown to improve ex vivo corneal wound healing [56].

So, taking all this into account, to minimize possible toxic effects on the ocular surface, recent guidelines [1, 2, 10, 17] for the management of allergic conjunctivitis recommend preservative-free eye drops whenever possible.

Other excipients present in eye drop formulations may lead to allergic contact dermatitis and contribute to the development of other chronic diseases such as DED [10]. Some of these products are wool alcohols, thiomersal, and phosphate buffers.

### Benefits of Hydration

The use of artificial tears with viscosity-enhancing agents such as hyaluronic acid (HA) to provide hydration and lubrication at the ocular surface has been shown to improve wound healing and to prevent dryness [56]. In this respect, HA has gained widespread use in eye surgeries and the treatment of eye disease, as HA-based materials are well tolerated and show excellent biocompatibility [56].

The main properties of HA are lubrication and water retention, which facilitate increasing corneal wettability in patients with DED [57]. Preclinical and clinical studies have shown that artificial tears containing HA provide acute and long-term therapeutic benefits in DED, including enhancement of corneal epithelium healing, improvement of the ocular surface function [58], normalization of clinical parameters, and alleviation and reduction of DED symptoms (hyperaemia, conjunctival redness and corneal wettability) [57, 59]. The combination of HA with active pharmaceutical compounds may also increase their bioavailability due to the high viscosity of HA [60], and its protective effect against conjunctival dehydration may provide benefits [61]. In conclusion, co-formulation with HA has potential benefits.

### Product Features, Patient Preferences and Adherence

A real-life study revealed that daily treatment of OA and patients´ use of their treatment for OA barely conforms with guideline recommendations for its management. Topical ocular decongestants and corticosteroids were used by most patients, independent of their diagnosis and OA severity [62]. Furthermore, patients often self-medicate with OTC preparations containing preservatives, including BAK, which may exacerbate ocular surface symptoms [7] and cause toxicity [8, 63].

Adherence to topical ophthalmic treatment in eye conditions has been widely studied. Patient preference for an eye drop can often be a primary factor in determining the level of compliance and adherence to treatment [46]. Discomfort upon instillation (as produced by NSAIDs) and long regular dosing (as required for efficacy in treatment with mast cells stabilizers) are other causes that have been associated with a poorer treatment adherence [1]. At present, there is a large market interest in preservative-free products, particularly for patients who need daily eye drops for long periods. Single-dose or single-use containers, which do not need preservatives [50, 54], and some multi-dose [64], preservative-free ophthalmic solutions are now available thanks to innovative device design, making it possible for patient preferences to be considered. In our experience, some patients may prefer single-dose presentations, as they find them more convenient, however a multi-dose presentation has the benefit of reducing product waste, common in single-dose eye drops. In addition, patients requiring permanent or long-term topical eye treatment, namely glaucoma, have better treatment compliance with multi-dose delivery systems [65, 66], and this may be applicable to AC. Several single-dose eye drop formulations are available for allergic conjunctivitis [50, 54], in addition to the usual BAK-containing multi-dose solutions, and a new preservative-free multi-dose formulation of bilastine with sodium hyaluronate has been recently approved [64].Along with the dispensation format, approaches to improving treatment adherence include treatment tolerability [67] and dosing schedules, as complex drug dosing regimens have been cited as a significant barrier to patient compliance [68, 69]. Simplifying dosing can be achieved by prescribing treatments that require once- or twice-daily dosing rather than multiple daily doses [69]. Once-daily ophthalmic medications are now available for allergic conjunctivitis [31, 37, 38], and this could foster adherence and treatment compliance.

## Key Messages: Implications for Clinical Practice

After reviewing the present situation of AC and its management with topical treatments, we can summarize some take-home messages for the practitioner:

- Allergic conjunctivitis is prevalent and often underdiagnosed. A multidisciplinary approach may be of use in patients presenting with compatible symptoms and other frequent concomitant conditions, as correct diagnosis is key for initiating appropriate treatment.
- Management includes environmental, non-pharmacological, and pharmacological measures. The first step is to avoid allergen exposure, underlining the importance of performing an allergy study.
- Topical ophthalmic antihistamines or double-action drugs are first-line pharmacological treatment. They are all effective, but formulations, presentations, and dosing may influence the outcomes.
- Preservatives in the formulation of ophthalmic solutions induce ocular surface damage and exacerbate SAC and PAC and may lead to other medical conditions such as DED. Therefore, the use of preservative-free ophthalmic solutions is advisable.
- Co-formulation with hyaluronic acid has potential benefits, as it has been shown to improve ocular surface wound healing and to prevent dryness, therefore protecting the ocular surface. It has also been shown to increase the ocular bioavailability of active drugs.
- Dosing is important when speaking of treatment compliance: studies indicate that the easier the dosing, the higher the adherence.
- Anti-allergic ophthalmic topical treatments are available in single-dose or single-use presentations, which do not need preservatives, and in multi-dose devices, preferably without preservatives.
- An innovative multi-dose, preservative-free, antihistamine formulation (with HA) for once-daily treatment of allergic conjunctivitis has been recently developed.

## Data Availability

No datasets were generated or analysed during the current study.
